# Functional analysis of brain derived neurotrophic factor (BDNF) in Huntington’s disease

**DOI:** 10.18632/aging.202603

**Published:** 2021-02-25

**Authors:** Zhike Zhou, Shanshan Zhong, Rongwei Zhang, Kexin Kang, Xiaoqian Zhang, Ying Xu, Chuansheng Zhao, Mei Zhao

**Affiliations:** 1 Department of Geriatrics, The First Affiliated Hospital, China Medical University, Shenyang 110001, Liaoning, PR China; 2 Department of Neurology, The First Affiliated Hospital, China Medical University, Shenyang 110001, Liaoning, PR China; 3 Computational Systems Biology Lab, Department of Biochemistry and Molecular Biology and Institute of Bioinformatics, The University of Georgia, Athens, GA 30602, USA; 4 Cancer Systems Biology Center, The China-Japan Union Hospital, Jilin University, Changchun, PR China; 5 Department of Cardiology, The Shengjing Affiliated Hospital, China Medical University, Shenyang 110004, Liaoning, PR China

**Keywords:** BDNF, Huntington's disease, differential expression, co-expression network

## Abstract

The aim of this study is to determine the molecular functions of brain derived neurotrophic factor (BDNF) in Huntington’s disease (HD). A total of 1,675 differentially expressed genes (DEGs) were overlapped from HD versus control and BDNF-low versus high groups. Five co-expression modules were constructed using weight gene correlation network analysis, among which the blue and turquoise modules were most strongly correlated with HD and low BDNF. Functional enrichment analyses revealed DEGs in these modules significantly enriched in GABAergic synapse, phagosome, cyclic adenosine monophosphate (cAMP), mitogen-activated protein kinase (MAPK), renin-angiotensin system (Ras), Ras-associated protein-1 and retrograde endocannabinoid signaling pathways. The intersection pathways of BDNF, such as cAMP, MAPK and Ras signaling pathways, were identified in global regulatory network. Further performance evaluation of low BDNF accurately predicted HD occurrence according to the area under the curve of 82.4%. In aggregate, our findings highlighted the involvement of low BDNF expression in HD pathogenesis, potentially mediated by cAMP, MAPK and Ras signaling pathways.

## INTRODUCTION

Huntington's disease (HD) is a monogenic neurodegenerative disorder mainly characterized by progressive cognitive impairment, motor dysfunction and psychiatric alterations [1]. The genetic cause of the disease is an expansion of CAG repeat in the mutant huntingtin (mHtt) gene, contributing to cortical atrophy and the preferential demise of medium spiny neurons in the striatum [2, 3]. A number of studies have shown that long-term survival of aforementioned neurons depends on the expression of brain derived neurotrophic factor (BDNF), which is reduced due to the mHtt-mediated mechanism in HD [4, 5]. Although there is no cure currently available for the disease, BDNF is thought to be an excellent therapeutic target for the clinical hallmarks of HD [6].

BDNF, encoding a member of the nerve growth factor family of proteins, is a crucial regulator of neuronal growth, differentiation and survival [7]. Previous evidence in mouse and cellular models of HD revealed that mHtt not only inhibited the synthesis and release of BDNF at cortico-striatal synaptic junction [8], but also disrupted its post-Golgi trafficking and vesicular transport [9, 10]. A resultant reduction in BDNF supply led to a failure of trophic support, which, in turn, exacerbated striatal degeneration and motor deficits [11, 12]. However, the neuropathological mechanisms of HD attributable to low BDNF expression remained elusive. Consequently, we sought to conduct a comprehensive bioinformatic analysis of BDNF based on gene expression data and functional annotations, which might gain insight into the molecular roles of BDNF underlying HD pathogenesis.

## RESULTS

### Identification of differentially expressed genes

**Figure 1:**
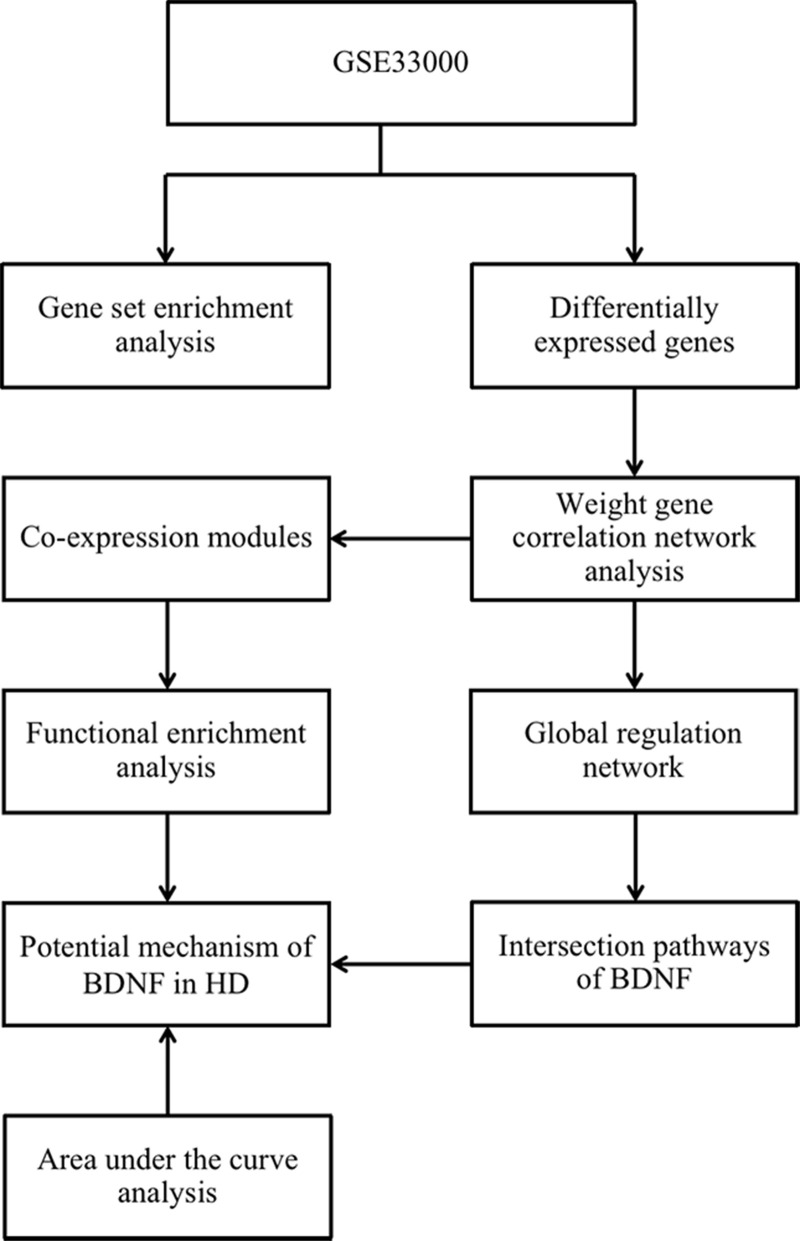
**The workflow diagram of the present study.** HD: Huntington’s disease.

**Figure 2:**
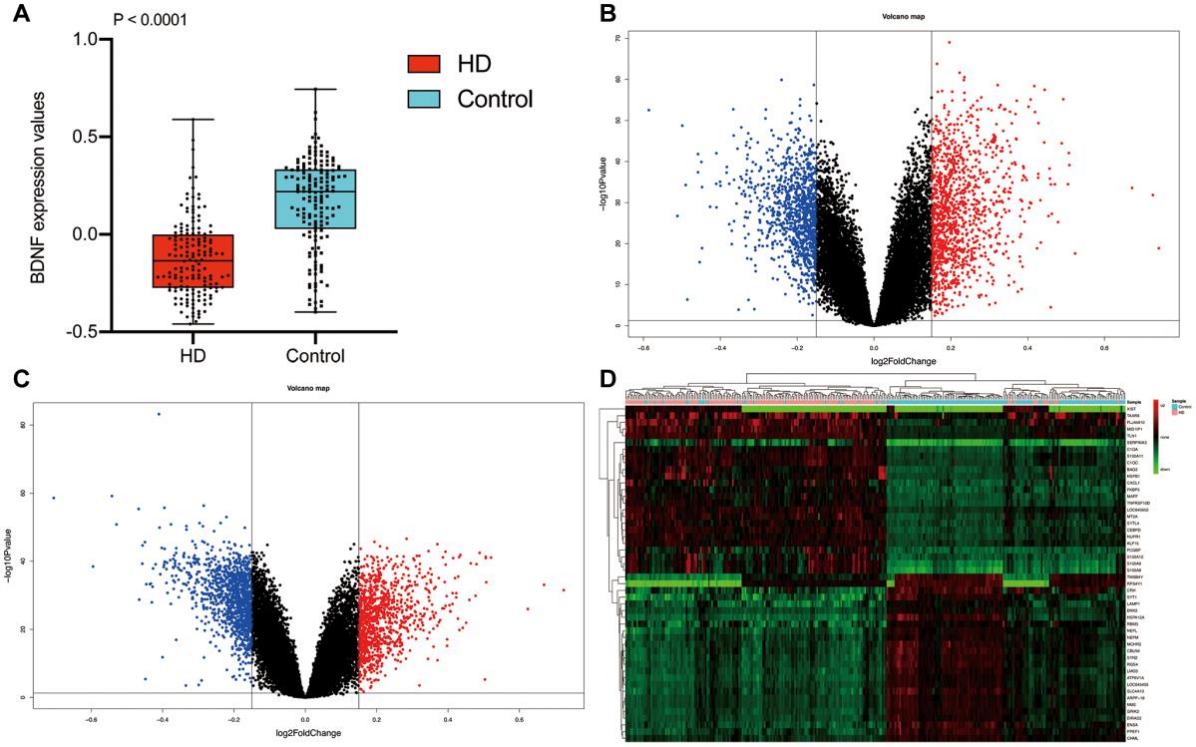
**Differential expression gene analysis.** BDNF expression between HD and non-dementia controls (**A**). Distribution of DEGs in the HD / control (**B**) and BDNF-low / high group (**C**): blue represents down-regulated and red indicates up-regulated. Heatmap of the top 25 down-regulated and up-regulated genes (**D**). HD: Huntington’s disease, DEGs: differential expression genes.

The workflow diagram of our study was presented in Figure 1. The mean ribose nucleic acid (RNA) expressions of BDNF in 157 HD cases (-0.12 ± 0.20) were significantly lower than that of 157 non-dementia controls (0.16 ± 0.23; P < 0.001) (Figure 2A). After the removal of repetitive and unannotated genes, 19,414 background genes were included for differentially expressed genes (DEGs) analysis. Significant changes in the expression of 2,294 genes (1,221 up-regulated and 1,037 down-regulated) were identified in HD versus non-dementia controls (Figure 2B); whilst 2,173 DEGs (1,028 up-regulated and 1,145 down-regulated) were determined in BDNF-low versus high group (Figure 2C). Thereafter, 1,675 overlapping DEGs were included between HD / control and BDNF-low / high cohorts. Heatmap of cluster analysis showed that the expression of the top 25 down-regulated and up-regulated DEGs distinguished HD from control samples (Figure 2D).

### Co-expression modules and functional enrichment analysis

**Figure 3:**
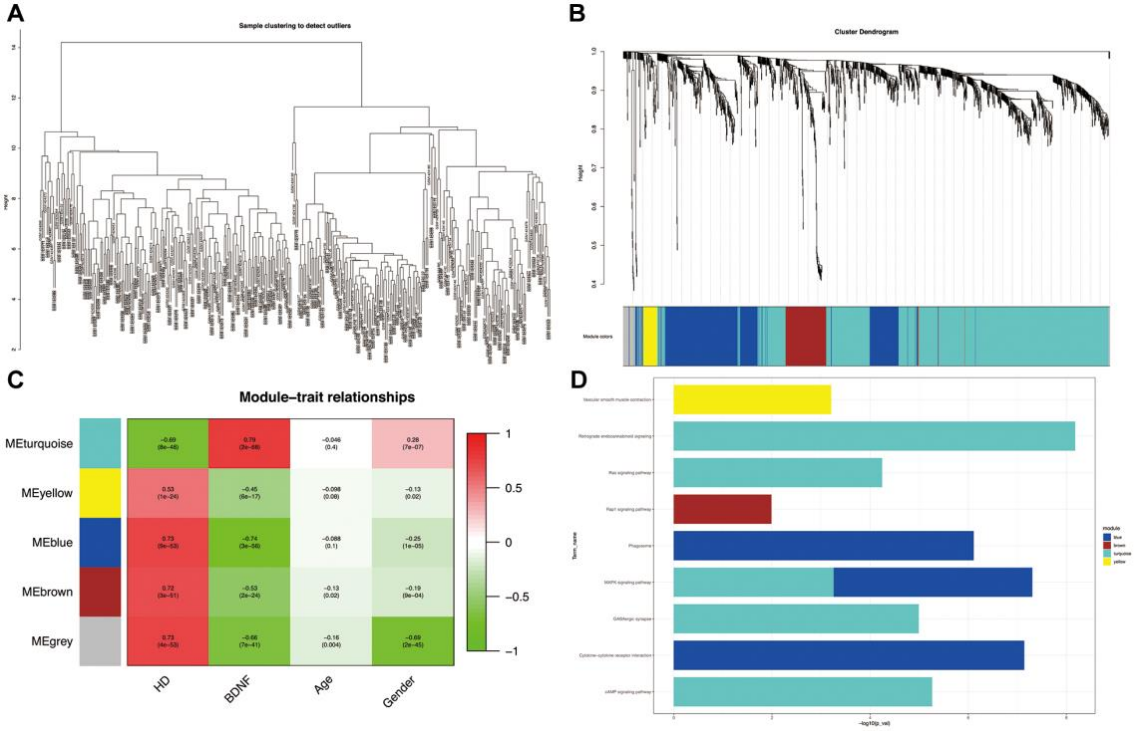
**Weighted correlation network analysis.** Plot of sample clustering (**A**). Cluster dendrogram of five modules and assigned module colors (**B**): grey indicates non-clustering genes. Heatmap of module-trait relationships (**C**): red indicates positively correlated and green represents negatively correlated. KEGG pathways of genes in co-expression module (**D**). HD: Huntington’s disease, KEGG: Kyoto Encyclopedia of Genes and Genomes.

All the samples passing the preset cut-off value (height = 15) belonged to specific clusters (Figure 3A). Five co-expression modules (blue, brown, gray, turquoise and yellow) were constructed by using WGGNA (Figure 3B). The heatmap of module-trait relationships (Figure 3C) revealed that the turquoise module had the strongest negative correlation with HD (correlation coefficient = -0.69, P = 8e-46) and the strongest positive correlation with BDNF expression (correlation coefficient = 0.79, P = 2e-68); whereas the blue module was the most positively correlated with HD (blue: correlation coefficient = 0.73, P = 9e-53) and the most negatively correlated with BDNF expression (blue: correlation coefficient = -0.74, P = 3e-56); likewise, the brown and yellow modules were positively correlated with HD (brown: correlation coefficient = 0.72, P = 3e-51; yellow: correlation coefficient = 0.53, P = 1e-24) and negatively correlated with BDNF expression (brown: correlation coefficient = -0.53, P = 2e-24; yellow: correlation coefficient = -0.45, P = 6e-17). As shown in Figure 3D, the DEGs of the turquoise module were enriched in KEGG pathways of GABAergic synapse, cyclic adenosine monophosphate (cAMP), mitogen-activated protein kinase (MAPK), renin-angiotensin system (Ras) and retrograde endocannabinoid signaling pathways; the blue module DEGs were involved in cytokine-cytokine receptor intersection, phagosome, and MAPK signaling pathways; the DGEs of brown and yellow modules participated in Ras-associated protein-1 (Rap1) signaling pathway and vascular smooth muscle contraction, respectively.

### Global regulation network and AUC analysis of BDNF

**Figure 4:**
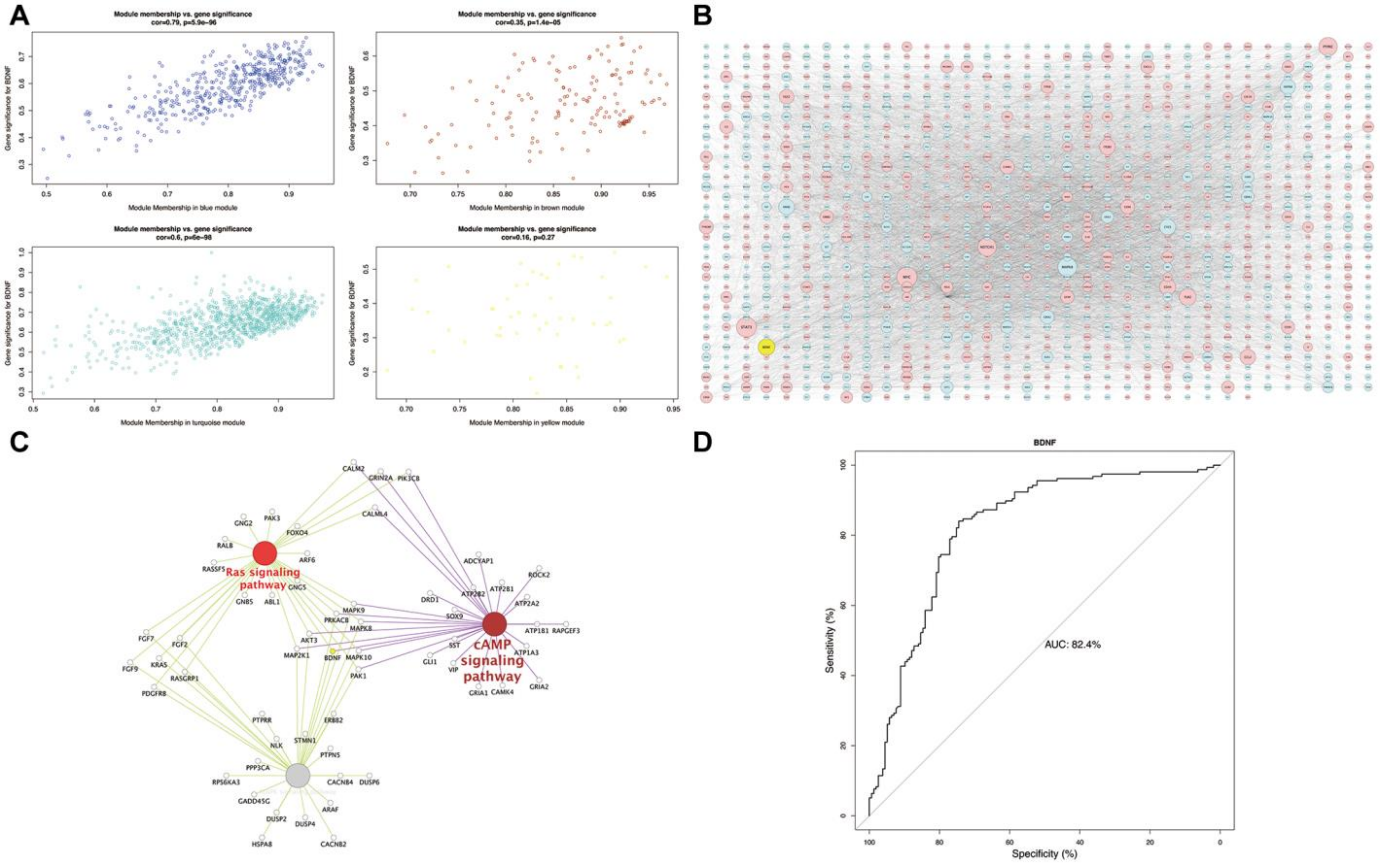
**Module-pathway regulatory network and AUC analysis.** Scatterplot of module membership vs. gene significance (**A**). Global regulatory network of blue and turquoise modules (**B**): red represents high expression; blue and yellow indicate low expression; node size indicates the degree of gene connectivity. Enrichment analyses of BDNF intersection pathways (**C**): yellow indicates the low BDNF expression. Performance evaluation of low BDNF in HD prediction (**D**). AUC: area under the curve, HD: Huntington’s disease.

The scatterplot of GS versus MM (Figure 4A) showed a strong correlation between intramodular connectivity and genetic phenotypes in the blue and turquoise modules (blue: correlation coefficient = 0.79, P = 5.9e-96; turquoise: correlation coefficient = 0.6, P = 6e-98). In the global regulation network (Figure 4B), low expression of BDNF interacting with DEGs was presented. Functional enrichment analysis identified the intersection pathways of BDNF, and all the genes enriching in cAMP, MAPK and Ras signaling pathways were exhibited in Figure 4C. The AUC analysis presented an accurate performance of low BDNF expression in predicting HD (AUC = 82.4%) (Figure 4D).

### Verification of BDNF-mediated pathways and the biological processes of GSEA

**Figure 5:**
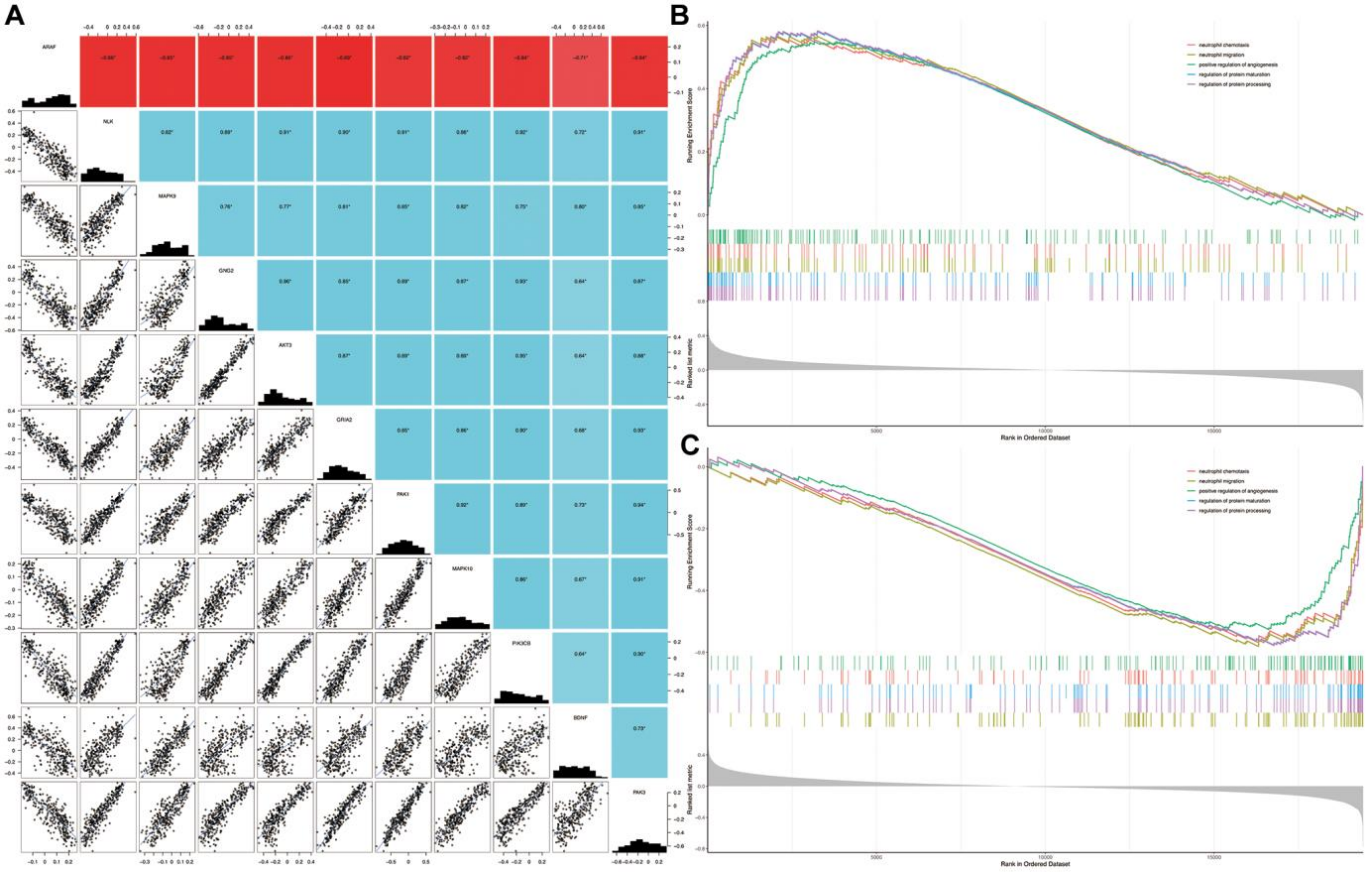
**Correlation among genes and gene set enrichment analysis.** Correlation of BDNF with signature genes (**A**): *P < 0.05; red indicates negative correlation; blue represents positive correlation. Biological processes enriched in HD (**B**) and BDNF-low (**C**) groups. HD: Huntington’s disease.

Five signature genes of each intersection pathway were listed in Supplementary Table 1. As shown in Figure 5A, the expression of BDNF were significantly positively or negatively correlated with each of the signature genes (P < 0.05). Compared with the non-dementia controls, the significantly enriched biological processes in HD were mainly related to neutrophil chemotaxis, neutrophil migration, positive regulation of angiogenesis, regulation of protein maturation and processing (Figure 5B). Similarly, biological processes of neutrophil chemotaxis, neutrophil migration, positive regulation of angiogenesis, regulation of protein maturation and processing, were significantly enriched in BDNF-low group (Figure 5C).

## DISCUSSION

In this study, we identified BDNF as a target gene of HD and the RNA data revealed that BDNF expression was down-regulated. The GSEA analyses engaging 19,414 background genes showed that DEGs in HD / control and BDNF-low / high groups were significantly enriched in regulation of angiogenesis, protein maturation and protein processing. Pathologically, the accumulation of N-terminal mHtt was observed to be responsible for protein processing impairment, protein misfolding and neuronal degeneration [13, 14]. It was worth noting that these biological processes were potentially related to HD as well as the low BDNF expression. Thenceforth, the global regulatory network and co-expression modules of DEGs interacting with BDNF were constructed to investigate the genome-level pathogenesis of BDNF in HD.

The results emerging from co-expression network analysis demonstrated that the blue and turquoise modules had the greatest correlation with HD and BDNF expression, in which the DEGs were involved in GABAergic synapse, phagosome, Rap1, cAMP, MAPK, Ras and retrograde endocannabinoid signaling pathways. Additional experiments from animal HD models linked the MAPK signaling pathway to the neurotoxicity of mHtt [15–17]. More specifically, the role for MAPK signaling in HD was supported by mHtt-induced inhibition of fast axonal transport through the activation of MAPKs, which provided a molecular basis for HD neuropathology [18]. An increase in BDNF was sufficient to preserve synaptic vesicle proteins and facilitate behavioral recovery in post-stroke mice, partially via MAPK signaling [19]. Several indirect evidences confirmed the linkage between BDNF and MAPK signaling, namely, the targeted deletion of MAPK kinases inhibited the neuroprotective action of BDNF, leading to neuronal apoptosis and brain developmental defects [20–22]. On the other hand, pretreatment with BDNF prevented MAPK phosphorylation activated by amyloid-beta peptide in the entorhinal cortex of Alzheimer’s disease, suggesting a negative correlation of BDNF with MAPK signaling [23]. Similarly, our results supported the likelihood that low expression of BDNF was involved in the hyperactivation of MAPK signaling and that enhancing BDNF expression could be neuroprotective in HD.

With except of the MAPK signaling, enrichment analysis of intersection pathways revealed that BDNF jointly participated in Ras and cAMP signaling pathways. The involvement of Ras has been increasingly reported in the pathophysiology of neurodegenerative diseases, such as Alzheimer’s disease [24] and HD [25]. Angiotensin converting enzyme (ACE) is a dipeptidase that belongs to the Ras and cleaves angiotensin I to generate angiotensin II (Ang II) [26]. In mouse models of Alzheimer’s disease [27] and HD [25], ACE inhibitors and Ang II antagonists were administrated to improve cognitive impairment by reducing mitochondrial oxidants.

Recently, the protective effects of candesartan (an inhibitor of Ang II) on BDNF loss and neuronal apoptosis has also been demonstrated in cognitively impaired rats, pointing to the reduction in BDNF on Ras signaling as a mechanism of neurodegeneration [28]. Moreover, BDNF participation in Ras / MAPK signaling pathways was extended by binding to and activating the tyrosine receptor kinase B, giving rise to the differentiation and survival of knock-in striatal cells in HD [29]. For cAMP signaling, it is essential for mHtt-induced energy metabolism deficits, especially in early stage of HD [30]. There was convincing evidence that inhibition of cAMP expression and mitochondrial respiratory chain dysfunction emerged in HD brain as early as 12 hours following mHtt transgene induction [31]; intriguingly, the administration of forskolin to raise cAMP levels attenuated the neurotoxicity of mHtt [32]. In addition, experiment from primary cultures of hippocampal neurons showed the deteriorative damage of cAMP signaling with the down-regulation of BDNF [33], which was in line with our findings on the involvement of cAMP signaling in low BDNF-mediated HD pathogenesis.

Further scatterplot of the relationship between MM and GS confirmed that DEGs in the blue and turquoise modules were strongly interacting with the BDNF expression. Based on these DEGs, the global regulatory network was constructed to predict the intersection pathways of BDNF, which supported the potential roles of BDNF reduction in HD pathophysiology via MAPK, Ras and cAMP signaling pathways. Owing to the low expression of BDNF, the susceptibility of these presented pathways to defects might be obvious, resulting in the development of HD under a variety of pathogenic mechanisms [29, 34]. The AUC analysis exhibited a good diagnostic performance of low BDNF in differentiation of HD cases from non-dementia controls, implying BDNF to be a potential biomarker of HD. It was consistent with previous animal experiment that low expression of BDNF was found in pre-symptomatic HD, and this pathological decline could be up-regulated through beneficial interventions, such as wheel operation and environmental enrichment [35]. Moreover, the analyses of Pearson correlation showed significant correlation of BDNF with signature genes, which suggested that alterations in BDNF expression led to changes in signature genes of each intersection pathway, thus providing computational statistical evidence that low BDNF expression related HD pathogenesis was mediated via cAMP, MAPK and Ras signaling pathways. Further investigation *in vivo* or *in vitro* is expected to verify the relevant pathways proposed in this study underlying pathological process of HD.

## CONCLUSIONS

Overall, we may presumptively declare that gene expression profiling is a promising approach to elucidate molecular roles of targeted gene in the HD occurrence. On the basis of our findings, BDNF is found to be down-regulated in HD, and its detrimental effects of low expression in the pathogenesis of HD might be mediated by MAPK, Ras and cAMP signaling pathways.

## MATERIALS AND METHODS

### Data resources

Rosetta / Merck Human 44k microarray analyses of postmortem prefrontal cortex samples were performed with RNA extracted from 157 HD patients and 157 age- and gender-matched controls in the GSE33000 dataset of Gene Expression Omnibus (GEO, https://www.ncbi.nlm.nih.gov/geo/) database [36]. A gene corresponding to multiple probes eliminated those with low expression and retained the highest one. The normalization processing on the gene expression data was conducted using normalizeBetweenArrays function in the limma package of R software version 3.6.2 [37].

### Differential expression analysis

Taking the mean expression value of BDNF to be cut-off point, the included samples were divided into BDNF-low and high groups. To identify differentially expressed genes (DEGs) in HD / control and BDNF-low / high cohorts, we computed empirical Bayes moderated t-statistics using lmFit and *eBayes* functions in limma packages. False discovery rate (FDR)-adjusted P < 0.05 and logarithm fold change (logFC) > 0.15 were considered statistically significant in the analysis of DEGs [37, 38].

### Co-expression network analysis

The expression data of DEGs overlapped from HD versus control as well as BDNF-low versus high groups were extracted to perform weight gene correlation network analysis (WGCNA). The hclust function was implemented to draw the clustering dendrogram eliminating the outliers of samples. Using the default unsigned network type, a soft thresholding power of 14 meeting the scale-free topology criterion was selected in the pickSoftThreshold function [39]. The WGCNA package was implied to predict the co-expression modules for assigning different color labels [40]. The minimum size of module was set to 30 genes to avoid small modules and guarantee separation. Functional annotations and enrichment analyses were conducted using the clusterProfiler package to screen genes enriched in Kyoto Encyclopedia of Genes and Genomes (KEGG) pathways. The FDR < 0.05 was considered as enrichment with statistical significance.

### Construction of global regulatory network and intersection pathways of BDNF

The scatter diagram of the module membership (MM) and gene significance (GS), respectively representing intramodular connectivity and genetic phenotype, was plotted using the verboseScatterplot function [41]. We selected the modules with the strongest positive or negative correlation with phenotypes to construct the global regulatory network in the STRING database (Search Tool for the Retrieval of Interacting Genes, https://www.string-db.org/) [42]. The visualization of global regulatory network and BDNF intersection pathways were accomplished by using the cytoscape software [43].

### Analysis of area under the curve (AUC)

The pROC function was utilized to evaluate the performance of target gene in distinction of HD and non-dementia. Receiver operating characteristic (ROC) curves exhibit the performance of dichotomies with sequential output, showing the sensitivity and specificity as output thresholds move into the range of all possible values [44]. ROC analysis is widely used in medical diagnostics, in which the performance of a classifier is measured by the area under the curve (AUC) [45]. An AUC value of 100% indicated complete prediction and 50% represented random selection. All P values were bilateral and statistical significance was set to the threshold less than 0.05.

### Signature genes for a pathway and gene set enrichment analysis (GSEA)

The quantified relationship of a gene with other genes was measured by correlation coefficient using Pearson correlation [46]. In term of each intersection pathway, we identified a small set of genes (the top 5) in the pathway as signature genes, whose expression showed the strongest correlation with other genes of the pathway [47]. A pathway was considered to be regulated or mediated by the target gene (i.e., BDNF) if the signature genes of the pathway were significantly correlated with target gene. The analysis of GSEA was conducted to filtrate the biological process of gene ontology terms that might be associated with HD and low BDNF [48, 49]. The default weight statistic was used for the permutation of 1000 times, and the threshold of significant enrichment was set as normalized P < 0.05. The enrichment data of GSEA analysis were visualized using ClusterProfler, ggplot2, enrichplot and GSEABase packages.

## Supplementary Material

Supplementary Table 1
