# Supplementary material for: Functional analysis of brain derived neurotrophic factor (BDNF) in Huntington’s disease
Source: Aging (Albany NY). 2021 Feb 25;13(4):6103–14. doi: 10.18632/aging.202603 (PMC7950280; doi:10.18632/aging.202603)
Supplement: Supplementary Table 1 [file aging-13-202603-s001.pdf]

SUPPLEMENTARY TABLE

Supplementary Table 1. Signature genes for each intersection pathway.

Pathway	Genes	Signature genes
cAMP signaling pathway	ADCYAP1, AKT3, ATP1A3, ATP1B1, ATP2A2, ATP2B1, ATP2B2, BDNF, CALM2, CALML4, CAMK4, DRD1, GLI1, GRIA1, GRIA2, GRIN2A, MAP2K1, MAPK10, MAPK8, MAPK9, PAK1, PIK3CB, PRKACB, RAPGEF3, ROCK2, SOX9, SST, VIP	AKT3, GRIA2, MAPK10, PAK1, PIK3CB
MAPK signaling pathway	AKT3, ARAF, BDNF, CACNB2, CACNB4, DUSP2, DUSP4, DUSP6, ERBB2, FGF2, FGF7, FGF9, GADD45G, HSPA8, KRAS, MAP2K1, MAPK10, MAPK8, MAPK9, NLK, PAK1, PDGFRB, PPP3CA, PRKACB, PTPN5, PTPRR, RASGRP1, RPS6KA3, STMN1	ARAF, MAPK9, MAPK10, NLK, PAK1
Ras signaling pathway	ABL1, AKT3, ARF6, BDNF, CALM2, CALML4, FGF2, FGF7, FGF9, FOXO4, GNB5, GNG2, GNG5, GRIN2A, KRAS, MAP2K1, MAPK10, MAPK8, MAPK9, PAK1, PAK3, PDGFRB, PIK3CB, PRKACB, RALB, RASGRP1, RASSF5	AKT3, GNG2, PAK1, PAK3, PIK3CB
